# [Retracted] Baicalin inhibits influenza virus A replication via activation of type I IFN signaling by reducing miR-146a

**DOI:** 10.3892/mmr.2025.13747

**Published:** 2025-11-10

**Authors:** Rui Li, Lianxin Wang

Mol Med Rep 20: 5041–5049, 2019; DOI: 10.3892/mmr.2019.10743

Following the publication of the above paper, it was drawn to the Editor's attention by a concerned reader that certain of the western blot data shown in Figs. 1C and 4C were strikingly similar to data that had already been published previously in articles submitted to other journals that were written by different authors at different research institutes. Upon assessing the data independently in the Editorial Office, some of the western blot data in Fig. 3C had also apparently been published previously in another article submitted to a different journal by different authors. Owing to the fact that the contentious data in the above article had already been published prior to its submission to *Molecular Medicine Reports*, the Editor has decided that this paper should be retracted from the Journal. The authors were asked for an explanation to account for these concerns, but the Editorial Office did not receive a reply The Editor apologizes to the readership for any inconvenience caused.

